# Protected Area as an Indicator of Ecological Sustainability? A Century of Development in Europe’s Boreal Forest

**DOI:** 10.1007/s13280-012-0375-1

**Published:** 2013-03-10

**Authors:** Marine Elbakidze, Per Angelstam, Nikolay Sobolev, Erik Degerman, Kjell Andersson, Robert Axelsson, Olle Höjer, Sandra Wennberg

**Affiliations:** 1 Faculty of Forest Sciences, School for Forest Management, Swedish University of Agricultural Sciences, PO Box 43, 730 91 Skinnskatteberg, Sweden; 2 Biodiversity Conservation Center, Moscow, Russian Federation; 3 Department of Aquatic Resources, Institute of Freshwater Research, Swedish University of Agricultural Sciences, 702 15 Örebro, Sweden; 4 Faculty of Forest Sciences, School for Forest Management, Swedish University of Agricultural Sciences, PO Box 43, 739 21 Skinnskatteberg, Sweden; 5 Valhallavägen 195, 106 48 Stockholm, Sweden

**Keywords:** Biodiversity, Conservation, Norway, Sweden, Finland, North West Russia

## Abstract

Protected area (PA) is an indicator linked to policies on ecological sustainability. We analyzed area, size, and categories of PAs in the European boreal forest biome in Norway, Sweden, Finland, and Russia from 1900 to 2010. The PA increased from 1.5 × 10^3^ ha in 1909 to 2.3 × 10^7^ ha in 2010. While the total PA in the boreal biome was 10.8 %, the figures ranged from 17.2 % in the northern, 7.9 % of the middle, and 8.7 % of the southern boreal sub-regions. The median size of PAs varied from 10 to 124 ha among countries. The categories of less strictly PAs increased over time. The proportion of area occupied by PAs is an important response indicator for conservation efforts. However, the use of PA as an indicator of ecological sustainability needs to consider ecosystem representation, functional connectivity and management categories.

**Electronic supplementary material:**

The online version of this article (doi:10.1007/s13280-012-0375-1) contains supplementary material, which is available to authorized users.

## Introduction

One tool to safeguard ecosystem services, thus addressing biodiversity conservation for ecological sustainability as expressed in international policies, is to establish effectively and equitably managed, ecologically representative, and well connected systems of protected areas (PAs) (CBD 2010a, b). The areal proportion of PAs is often used as one of the indicators to monitor the implementation of policies on ecological sustainability (Frank et al. 2007; Butchart et al. 2010). Three policy areas that exemplify this are biodiversity conservation (CBD 2011), sustainable forest management (Forest Europe 2011), and ecosystem services (Kumar 2010). In 2010, the Strategic Plan for Biodiversity 2011–2020 and the Aichi Targets were adopted at the meeting of the Conference of the Parties to the Convention on Biological Diversity (CBD 2010a, b, 2011). The 11th Aichi target aims at protecting by 2020 at least 17 % of terrestrial and inland water areas as functional habitat networks for biodiversity and ecosystem services, and 10 % of coastal and marine areas (CBD 2011).

In spite of many efforts globally, actions to reduce pressure on biodiversity have not been sufficient, and integration of biodiversity issues into broader policies, strategies, and actions as a response have not been appropriate (CBD 2010a, b). This is also reflected in many empirical studies looking at the impacts of policy implementation on ecological sustainability. For example, Butchart et al. (2010) showed that neither is the rate of biodiversity loss being reduced, nor is the pressure on biodiversity decreasing. Although the total area of PAs grows, little is known of the extent to which the current global PA network fulfills its goal of protecting biodiversity. The premise that a higher percentage of protected land is evidence for improved conservation is thus being questioned (Rodrigues et al. 2004).

The conservation of ecosystems’ composition, structure, and function (Noss 1990)—the foundation for delivery of ecosystem services and biodiversity conservation—involves the establishment, management, and restoration of functional habitat networks, including both PAs and their matrix (Craig et al. 2000). While biodiversity conservation has been monitored using comparisons among countries, ecoregions, or biomes of PAs expressed in percentages or as total area (Parviainen and Frank 2003), there have been only a few attempts to compare the relative conservation efforts made by different nations over time (e.g., Frank et al. 2007). This kind of evaluation requires a historical perspective on the development of PA in different countries located in the same ecoregion or biome using different indicators. Measurement of any indicator may relate to pressure upon biodiversity (resource consumption, overexploitation, and climate change impacts), state (extinction risk, habitat extent and condition, and community composition) or response (coverage of PAs, sustainable forest management, policy responses) (Rapport and Friend 1979; Butchart et al. 2010).

The aim of this paper is to analyze and compare the development over time of PA as one of the response indicators of ecological sustainability in Europe’s boreal forest regions and countries. Conservation of the boreal forest, the second largest biome in the world, has received limited attention from the international community (Dudley et al. 1998; Bradshaw et al. 2009). Being relatively remote from centers of economic development, the boreal forest is the least affected by exploitation and use among the European ecoregions (Hannah et al. 1995; Angelstam et al. 2013). There is therefore still an opportunity to achieve high levels of conservation for boreal ecosystems, which address ecological integrity and resilience (Angelstam et al. 2004a). Recently, also the global importance of boreal forest protection for mitigation and adaptation to climate change has been highlighted (Bradshaw et al. 2009; Carlson et al. 2009; Dise 2009). Currently, however, the pressure on boreal ecosystems is growing due to increasing interests in using wood, non-wood goods, and other ecosystem services for economic development (Olsen 1993; Dudley et al. 1995; Korpilahti et al. 1996). This use leads to an accelerating loss of intact forest landscapes (Yaroshenko et al. 2001), habitat fragmentation (Elbakidze et al. 2011), and altered ecosystem processes, all of which affect species and forest functions (Burnett et al. 2003; Bradshaw et al. 2009). Additionally, climate change creates new challenges for biodiversity conservation (Heller and Zavaleta 2009) in the boreal regions, where the climate warming will be globally most profound (Ruckstuhl et al. 2008).

While informal PAs have a long history in the forms of spiritual and sacred natural areas and forests managed for hunting, Sweden became the first country on the European continent to establish PAs by law (in 1909, see Wramner and Nygård 2010). This was in the boreal forest. We describe the development of PAs, in terms of size and management of PAs between 1909 and 2010 in the northern, middle, and southern boreal forest sub-regions in Norway, Sweden, Finland, and NW Russia, which together encompass Europe’s boreal forest. This comparative analysis can provide important input to a collaborative learning process within and among countries towards the implementation of internationally agreed policies on ecological sustainability. Finally, we discuss the need to complement the PA as a response indicator with indicators that also reflect the state of ecological sustainability.

## Materials and Methods

### Study Area

This study focuses on the European boreal forest, of which 99 % is located in Norway, Sweden, Finland, and the Russian Federation, and with the remainder in Scotland. As pointed out by Tukhanen (1980) climate is a key driver for the location of different ecoregions and biomes, which make them suitable as units for ecological monitoring. However, there are different schools of thought about the geographical location of the boreal forest biome (Tishkov 2002). For example, some scholars (Vorovyev 1953) include the hemiboreal transition zone between the boreal forest (sensu Ahti et al. 1968) and temperate deciduous forests within the boreal biome. The most common division of the boreal forest in Europe, however, excludes the hemiboreal sub-region, and divides the boreal forest into northern, middle, and southern sub-regions (Ahti et al. 1968; Mayer 1984; Bohn et al. 2004).

We chose Bohn’s et al. (2004) map of natural vegetation of Europe to define the borders of the boreal forest and its sub-regions as our study area. This map was produced by a team of international experts for the entire European continent at a scale of 1:2 500 000. A unified definition of the natural vegetation types, means for processing and designating the mapping units, and a systematic general legend for their classification were developed.

**Fig. 1:**
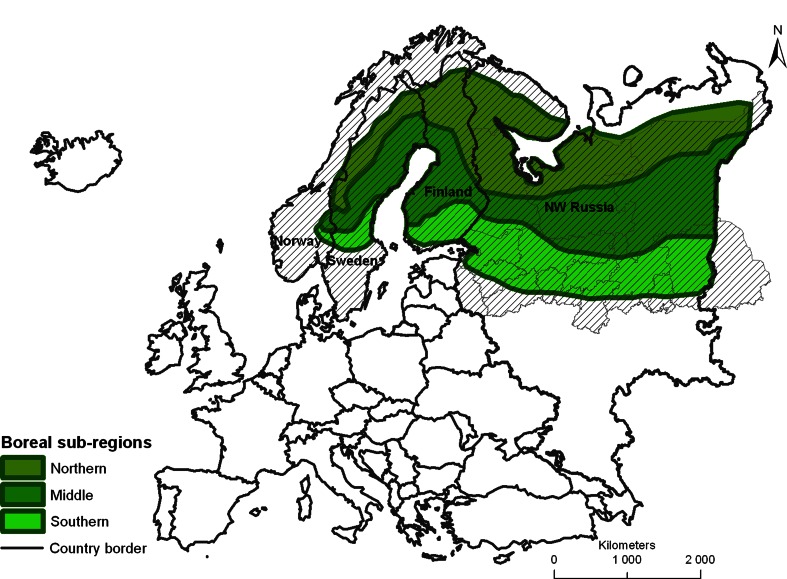
The location of the northern, middle, and southern boreal forest sub-regions in northern Europe

Following Bohn et al. (2004), our study area includes the boreal forest found from Fennoscandia in the west over the East European plain to the Ural Mountains (Fig. 1). The boreal forest includes different formations of coniferous and mixed deciduous-coniferous forests. These are divided into sub-regions according to species composition, differences in climate and site conditions that are in turn subdivided according to nutrient balance, altitudinal belts, water balance, and geographical location (Bohn et al. 2004), which have different disturbance regimes (Shorohova et al. 2011). The main sub-units of formation of boreal forest are presented in Table S1 (Electronic Supplementary Material) and Fig. 1.

### Analyses of PAs Over Time and in Space

Our analyses focused on terrestrial and inland water areas in the boreal forest biome formally protected during the period 1909–2010. We define PAs as those designated and managed under national nature conservation legislation and governmental conservation programs, including nature conservation acts of the entities of the Russian Federation (e.g., decrees, decisions, rules, regulations, orders, etc.) (Table S1). All selected PAs in each country were grouped based on their location in the northern, middle, and southern boreal forest sub-regions as defined by Bohn et al. (2004). The analysis of the development of PAs over time included two parts.

First, we analyzed the total area and area proportion of PAs as well as PA management categories during the last century in the European boreal forest biome as a whole, and its northern, middle, and southern sub-regions. Considering that PA categories vary among the four countries, we used the IUCN management categories (Dudley 2008) (Table 1) to harmonize the diversity of national categories. However, the status in IUCN-assignment varies between countries. Sweden made the IUCN categorization in 2002, however, since then a further 1600 nature reserves have been established, and a revision that take the new IUCN guideline (2008) into further consideration will take place. In Norway a simplified categorization has been done. In Finland and Russia the IUCN categories have not been officially assigned to individual PAs, and the matching of national and IUCN categories was based on non-official interpretations provided by the national agencies and experts. For each individual PA the most appropriate IUCN category was identified.

**Table 1:** Correlation between IUCN management categories and national categories of protected areas: *Ia* strict nature reserve, *Ib* wilderness area, *II* national park, *III* natural monument or feature, *IV* habitat/species management area, *V* protected landscape/seascape, *VI* protected area with sustainable use of natural resources (Dudley 2008)

IUCN	Norway	Sweden	Finland	Russian Federation
Ia	Nature reserve	Nature protection area	Strict nature reserve	Strict nature reserve
		Nature reserve		
Ib		Nature reserve	Wilderness reserve,	
			Old-growth forest reserve	
			Mire conservation reserve	
			Wilderness reserve	
			Nature conservation program	
			Nature conservation program site	
II	National park	National park	National park	Natural park
		Nature reserve	National hiking area	National park
			Nature conservation program	
III	Natural monument	National park		Natural and cultural area
		Nature reserve		Natural monument
IV	Botanical conservation area	National park	Herb-rich forest reserve	Nature reserve (federal and municipal)
		Nature reserve	Nature reserve (MH)	
	Wildlife conservation area	Nature protection area	Nature conservation program site	Municipal landscape reserve
				Municipal botanic reserve
				Peat deposit
	Protected landscape		Area designated in land use plan	Protected natural complex
				Protected bog
			Protected forest	Protected landscape
			Reserved sites	Natural monument
				Regional nature reserve
				Protected historic and natural complex
				Regional nature reserve
V	Protected landscape	Nature reserve	Recreation site (MH)	Regional nature reserve
		Nature protection area		Tourist and recreational area
				Recourse reserve
				Garden art monument
				Protected landscape
				Natural monument
VI				Regional nature reserve
				Natural monument
				Green zone
				Recreational area
				Hunting resource protection zone
				Therapeutic area
				Forest genetic reserve
				High value cranberry bog
				Crayfish nursery
				Protected historic and natural complex

Second, we compared PA development over time in Norway, Sweden, Finland, and NW Russia. The comparison included a statistical description of the average annual change in area proportion of PAs and PA sizes in different sub-regions of boreal forest in each of the four countries. The former was completed using linear regression of area proportion versus time with decade resolution, and the average annual change was expressed as the slope of the regression line. When comparing sizes of PAs using statistical analyses we transformed data using log_10_ to avoid a skewed data set.

### Materials

All PA data used in this study are official and provided to us by the responsible governmental organization for nature conservation in each country, or was extracted by us from the official web-sites of those organizations. The data on PAs in Norway was extracted from the Directorate for Nature Management in Norway (Table S1). For Sweden, the staff at the Swedish Environmental Protection Agency provided the complete data set on PAs located in the country’s boreal forest biome. The data about PAs in the northern and middle boreal sub-regions in Finland was provided by the Finnish Forestry and Natural Heritage Service (Metsähallitus in Finnish). For the southern boreal forest sub-region in Finland we extracted data from European Common Database on Nationally Designated Areas (National CDDA) (Table S2). The data on PAs in NW Russia was gathered using a broad range of sources. First, the legal and official documents on PA’s designation found on the web-sites of federal, regional, and municipal authorities and PA administrations were analyzed. Second, we used reviews on territorial conservation history and development in the Russian Empire and the former USSR (Table S1).

The collected data on PAs in Europe’s boreal forest biome were organized into a database that included name; national designation to a particular category as defined in Table 1; location (northern, middle, or southern boreal sub-region); size in hectares; year of designation, and year of conversion of a PA to other type of PA or unprotected area; and IUCN management category. For this study we identified a total of 17 086 PAs.

## Results

### Protected Areas Over Time in Europe’s Boreal Forest

**Fig. 2:**
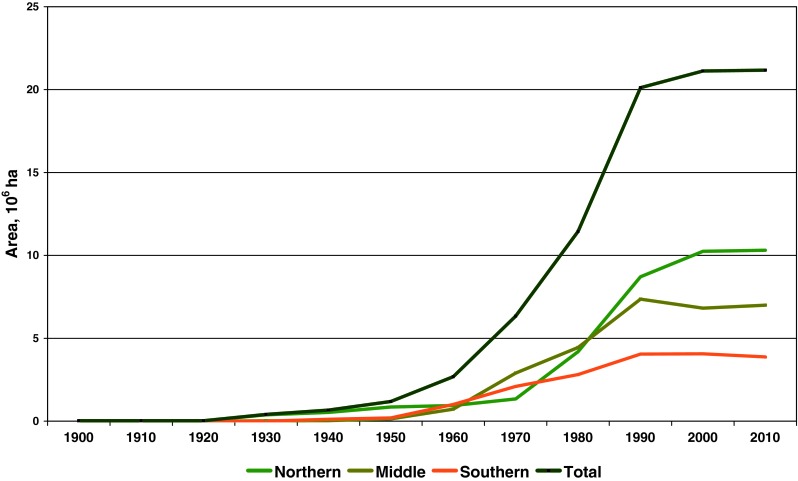
Cumulative growth of PAs in Europe’s northern, middle, and southern boreal forest sub-regions by decade

The area extent of PAs in Europe’s boreal forest biome increased steadily from 1909 to 2010. However, there was considerable variation in the dynamics among different decades and northern, middle, and southern boreal sub-regions (Fig. 2). After the establishment of the first PAs in the boreal forest biome in 1909, during the next two decades there was no further development. The growth started in the 1930s, and during the following 10 years the total area of PAs increased 12-fold (from approximately 35 000 to 410 000 ha) due to the rapid growth the extent of PAs in the northern boreal sub-region. The total area of PAs reached one million ha in the 1950s, and almost 70 % of this area was located in the northern boreal sub-region. A clear jump in growth happened since the 1960s when the total area of PAs increased from 2.7 million ha to more than 20 million ha during the following four decades. The steepest growth took place in the 1990s when the total area increased with 8.7 million ha during one decade. However, during the past decade the growth of PAs slowed down, especially in the middle sub-region where the total area of PAs even decreased (Table 2).

**Table 2:** The area proportion (%) of PAs in northern (N), middle (M), and southern (S) boreal sub-regions in Norway, Sweden, Finland, and NW Russia, and in the four countries together during different decades since the 1990s

	Norway			Sweden			Finland			NW Russia			Total		
	N	M	S	N	M	S	N	M	S	N	M	S	N	M	S
1900–				0.4									0.1	0.0	00
1910–				0.4									0.1	0.0	0.0
1920–				0.4									0.1	0.0	0.0
1930–		0.1		0.4						0.8			0.6	0.0	0.0
1940–		0.1		1.0	0.1					1.1		0.3	0.8	0.0	0.2
1950–		0.1		1.0	0.1		3.1	0.1	0.7	1.1	0.2	0.3	1.4	0.2	0.3
1960–	0.1	0.1		1.0	0.1		3.1	0.1	0.7	1.4	1.1	2.3	1.6	0.8	1.8
1970–	0.2	0.1	0.3	3.3	0.1	0.8	3.1	0.1	0.8	2.1	4.6	4.9	2.4	3.2	3.7
1980–	1.1	3.9	1.1	7.9	0.6	1.5	9.0	1.8	1.3	6.9	6.6	6.6	7.2	4.9	5.0
1990–	1.9	4.3	1.8	13.0	2.1	1.8	18.1	2.6	2.7	12.4	9.3	9.5	13.0	7.0	7.3
2000–	16.3	12.7	4.4	25.2	2.9	2.2	21.3	2.6	4.4	13.3	8.1	9.7	16.2	6.5	7.6
2010–	18.9	13.7	5.3	25.4	3.2	2.4	21.3	2.6	4.4	14.6	10.1	11.9	17.2	7.9	8.7

**Fig. 3:**
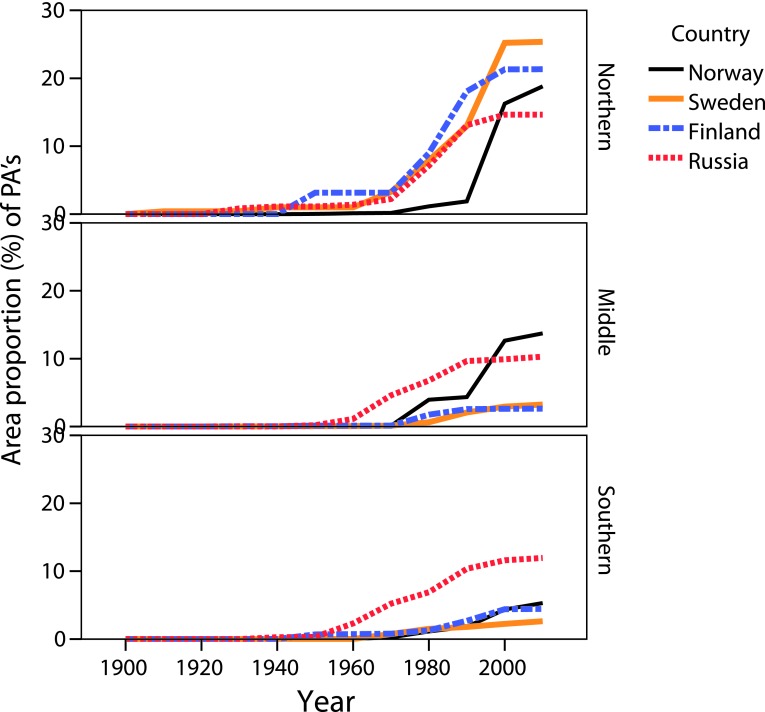
Cumulative growth of the area proportion of PAs in northern, middle, and southern boreal forest sub-regions in Europe

During the first four decades the growth in the total area of PAs took place only in the northern boreal forest (Fig. 3; Table 2). From the 1960s and during the next three decades there were almost no differences in extent of PAs among all three boreal sub-regions. However, from the 1990s onward the area proportion of PAs became again much higher in the northern boreal forest (Table 2).

**Fig. 4:**
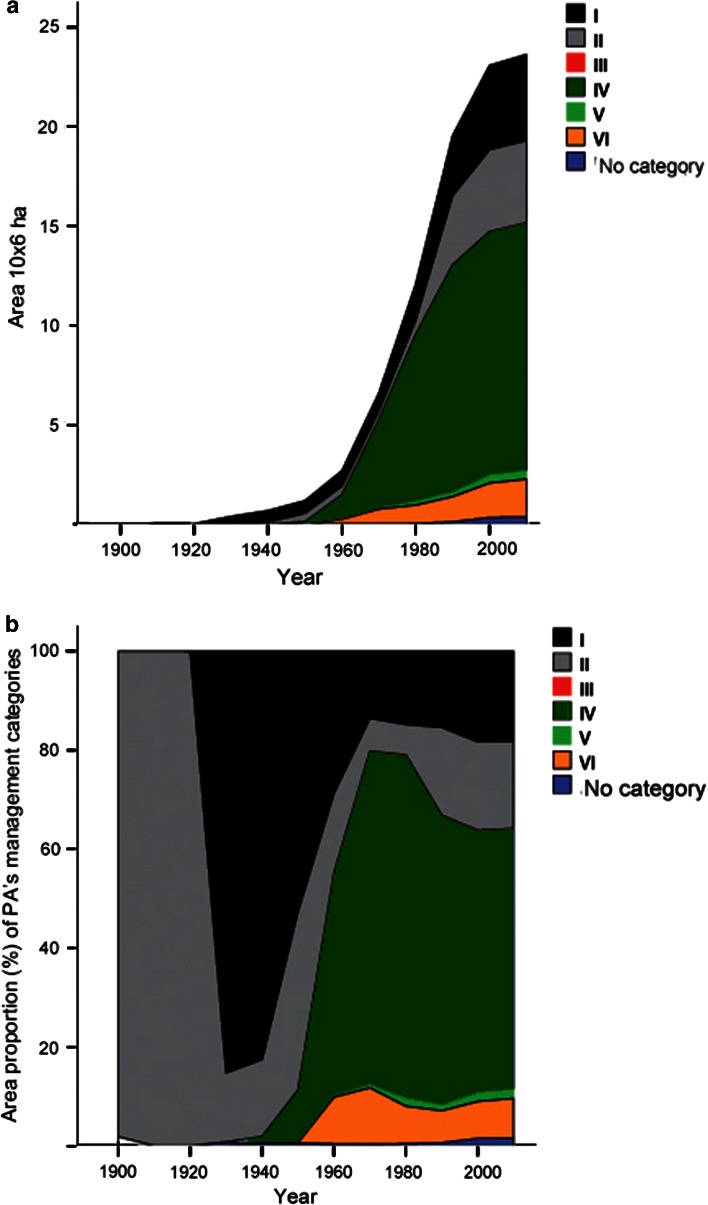
Dynamics of IUCN protected area management categories in Europe’s boreal forest over time: **a** total area of IUCN management categories over time; **b** dynamics of area proportion of each IUCN management category (for the names of IUCN management categories see Table 1)

Strict nature reserves (IUCN category I), national parks (IUCN category II), and those analogous to today’s habitat management areas (IUCN category IV) were the main categories of PAs designated between 1909 and 1920 (Fig. 4a, b). They made up almost 99 % of total area of PAs in Europe’s boreal forest biome. Their area proportion began decreasing in the 1930s, and sharply declined in the 1960s when the other PA management categories developed. The area proportions of the current PA management categories have been relatively stable since the 1990s. Since the 1970s the habitat/species management areas (IUCN category IV) had been the most widespread PA category, and occupied in total the largest area in comparison with others PA categories (Fig. 4a, b). At the same time, the actual area occupied by each PA category has been steadily growing over the last 100 years, even if their area proportion varied during different decades (Fig. 4a).

**Fig. 5:**
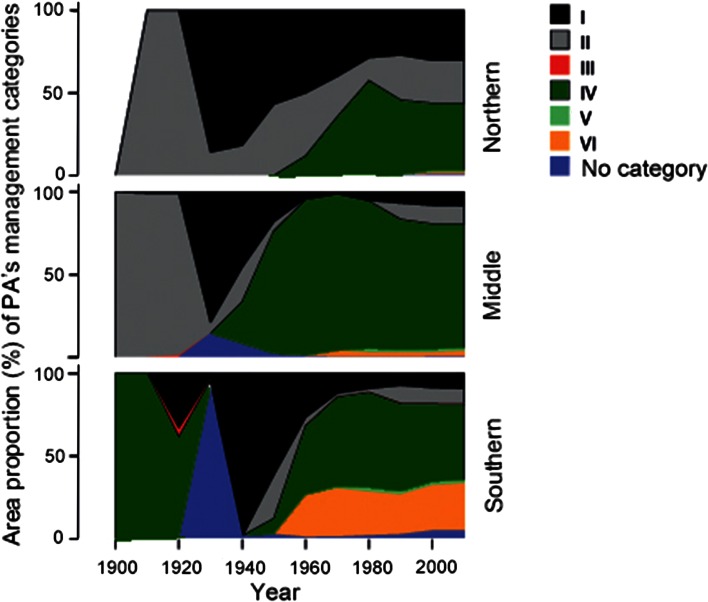
Dynamics of area proportion of IUCN management category in the northern, middle, and southern boreal forest sub-regions in Europe (for the names of IUCN management categories see Table 1)

There were considerable differences in the profile of PA management categories among the northern, middle, and southern boreal sub-regions over the last 100 years (Fig. 5). The development was, from more strictly protected categories (I and II) in the north, to less strict management categories in the middle and southern boreal forest.

### Comparisons Among Countries

The four countries included in this study began their PA development in different decades. Sweden was the first country to establish PAs in Europe’s boreal forest biome. This took place in the northern sub-region, and during the following four decades it was the only sub-region where PAs were established. From the 1960s, PAs appeared in all three boreal sub-regions in Sweden.

Norway and the Russia Federation were the next two countries to establish PAs in the European boreal forest. This process began in the 1930s. In Norway, PAs were created first in the middle boreal forest and their area proportion was stable during the next two decades. In the 1960s PAs appeared in the northern sub-region, and since the 1970s PAs have been established in all Norwegian boreal forest sub-regions (Fig. 3; Table 2). The middle boreal sub-region had the largest area proportion of PAs between 1980 and 2000. Northern boreal sub-region has come into focus for PAs’ development since the 2000s, after which the total area of PAs increased faster (Table 2; Fig. 3).

In NW Russia, PA development began in the northern boreal sub-region, and in the 1940s PAs appeared in the southern sub-region. By the 1950s PAs had been established in all boreal sub-regions. The southern boreal sub-region was in focus for PAs’ development during three decades since the 1960s. From the 1990s onward, the focus shifted to the northern forests where the area proportion of PAs subsequently became higher than in the other two sub-regions.

In Finland (within the country’s present border), the PA development began in all three sub-regions in the 1950s. After almost three status quo decades, since the 1980s the cover of PAs had increased in the northern boreal forest and this sub-region was favored during the next decades.

In conclusion, while the area of PAs has grown steadily in each country over the past century, at the same time three different patterns of PA development can be distinguished since the 1950s: (1) Rapid growth when the area proportion of PAs increased more than three times from one decade to the next. This happened once in Sweden and Norway in the northern and middle sub-regions, respectively. (2) No change in the area proportion of PAs for several decades. This occurred once in Norway in the middle sub-region; three times in Sweden (one time in northern, and twice in the southern sub-region); six times in Finland (twice in each sub-region in different decades). (3) Decrease in the area extent of PAs from one decade to the next. This happened once only in Russia in the middle boreal sub-region (Table 2).

The average annual change in the area proportion of PAs during the past century was different in different sub-regions in the four countries. In Norway, Sweden and Finland the average growth was uneven among sub-regions with faster growth in northern boreal forest in Sweden and Finland, and in the middle boreal sub-region in Norway. Since the 1950s the maximum average annual increase (0.6 %) occurred in the middle boreal forest in Norway and in the northern sub-region in Sweden. In Russia the average annual growth was more or less even among sub-regions and varied from 0.2 to 0.3 % (Table 3).

**Table 3:** The annual change (in %) of the increase of total proportion of PAs in the boreal forests in Norway, Sweden, Finland, and the European part of the Russian Federation

Country	Boreal forests	Total area proportion (%) of PAs			Average annual change (%) of total PAs	
		In 1909	In 1950	In 2010	1909–1950	1950–2010
Norway	Northern	0	0.0	18.9	<0.001	0.3
	Middle	0	0.1	13.7	0.003	0.6
	Southern	0	0.0	5.3	<0.001	0.1
Sweden	Northern	0.3	1.0	32.6	0.015	0.6
	Middle	0.0	0.0	6.1	<0.001	0.1
	Southern	0.0	0.0	5.5	<0.001	0.1
Finland	Northern	0	3.1	21.3	0.045	0.4
	Middle	0	0.1	2.6	0.003	0.1
	Southern	0	0.7	4.4	0.010	0.1
Russia	Northern	0	1.1	14.6	0.028	0.3
	Middle	0	0.2	10.3	0.003	0.2
	Southern	0	0.3	11.9	0.007	0.2

**Fig. 6:**
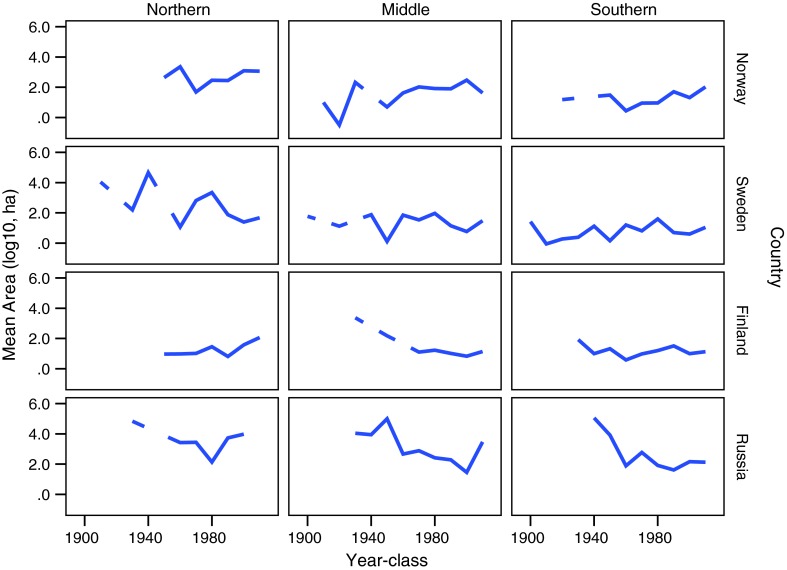
The average size of PAs (log_10_, hectares) in Norway, Sweden, Finland, and NW Russia and in northern, middle, and southern boreal forests during 1900–2010 (decades)

**Fig. 7:**
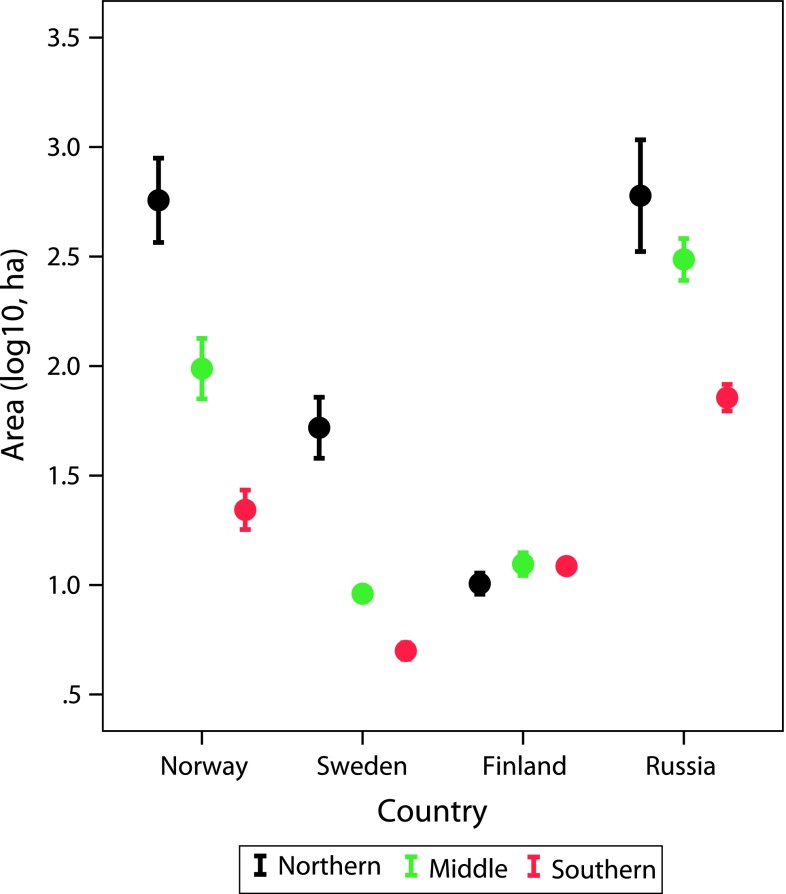
Average size of PAs (log_10_, hectares) with 95 % confidence intervals in Norway, Sweden, Finland, and NW Russia and in northern, middle, and southern boreal forests during 1900–2010 (decades)

The median size of PAs was 48 ha in Norway, 64 ha in Sweden, 10 ha in Finland, and 124 ha in Russia. The average size of PAs did not change significantly over time since the 1900s in the three sub-regions in Norway and Sweden (Figs. 6, 7). In Finland the average size declined significantly among decades in the middle boreal sub-region (Pearson correlation −0.892, *p* = 0.007, *n* = 7), tended to increase in northern sub-region (Pearson correlation 0.735, *p* = 0.06), whereas there was no significant correlation in the southern boreal region. In Russia there were trends of decreasing sizes of PAs, but only for the middle sub-region the correlation was significant (−0.752, *p* = 0.031, *n* = 8). The negative trend in Russia was due to a large PA being established early during the study period. From the first decade of the twentieth century and during the following four decades the median size of PAs in Russia was 61 776 ha (*n* = 4), whereas the median in the other countries was 12 ha (Norway), 54 ha (Sweden), and 161 ha (Finland). In all countries, except Finland, the average size of PAs was larger in the north as compared to the middle and southern sub-regions (Figs. 6, 7).

## Discussion

### Protected Area as a Response Indicator

This study demonstrates that the areal extent of PAs in the boreal forest biome increased from approximately 0.0015 million ha in 1909 to 23 million ha in 2010. Most of this increase took place since the 1980s onward. We also show that the area proportion, size, and management profiles of PAs were very different over time among boreal sub-regions and countries.

Throughout this 100-year study period, the northern boreal forest sub-region with the least productive forest ecosystems was preferentially protected. As a result, while in the four studied European countries by the end of 2010 the overall area proportion of PAs was 10.8 % of the total boreal forest biome, the figures ranged from 17.2 % of the northern, 7.9 % of the middle, and 8.7 % of the southern boreal forest sub-regions. Our study thus confirms the conclusion made by Gaston et al. (2008) that PA development has resulted in ‘a bias towards including large, contiguous areas of land of limited economic value in PA systems’. The uneven representation of PAs among Europe’s boreal forest sub-regions, and among the studied countries that was maintained during almost the entire previous century presents a big challenge for boreal forest conservation (e.g., Hanski 2011; Uotila et al. 2002; Virkkala and Rajasärkkä 2007).

Another challenge for ecological sustainability is that the vast majority of boreal PAs are small, with the smallest areas in the southern boreal sub-region. According to many studies concerning the requirements of species with different life histories (McNab 1963; Belovsky 1987; Menges 1991; Meffe and Carroll 1994; Edenius and Sjöberg 1997; Jansson and Angelstam 1999; Biedermann 2003; Jansson and Andrén 2003; Angelstam et al. 2004b; Roberge and Angelstam 2004; Linnell et al. 2005), it is evident that the sizes of the many of PAs have not been and are not sufficient for focal and umbrella species such as specialized birds and area-demanding mammals.

Regarding PA management, this study shows that the PAs belong to several different categories. However, the extent to which these categories are adapted to the regional context in Europe’s boreal biome in order to deliver desired ecosystem services remains to be studied. At present there is limited correspondence among the national categories of PAs and IUCN management categories in the four studied countries. There is no clear and globally consistent alignment between the IUCN categories and their application (Leroux et al. 2010).

Summing up, the area proportion of PAs is an important response indicator for conservation efforts. However, obviously, it needs to be combined with other relevant indicators, because the area proportion of protection of a region does not necessarily mean that PA networks are in place in terms of providing functional habitat networks for different ecosystems, or for other dimensions of ecological sustainability. We thus agree with Chape et al. (2005) who wrote ‘the setting of minimum percentage targets for biodiversity conservation of biomes or ecoregions may create political comfort but does not provide a basis for realistic assessment’, and ‘measurements of numbers and extent must be combined with assessment of conservation effectiveness to achieve meaningful results’.

### Protected Area as an Indicator for Ecological Sustainability?

To improve the use of PA as an indicator for ecological sustainability representation of boreal forest ecosystems, functionality of the network of PAs, the management of PAs, and the qualities of the surrounding matrix have to be considered.

First, one has to consider that the ecosystems and habitats vary among different boreal forest sub-regions and countries (Shorohova et al. 2011). Sufficient representation of ecosystems with different disturbance regimes in PA networks (Angelstam and Kuuluvainen 2004; Shorohova et al. 2011) is thus crucial for the conservation of species, habitats, and processes (Brumelis et al. 2011).

Second, for the conservation of species in the boreal forest biome, the functionality of the network of PAs of a particular ecosystem type needs to be assessed individually. The use of spatial modeling of the size, quality, and juxtaposition of PAs can be used to assess of the functionality of different networks (Andersson et al. 2012). Several studies show that the functionality of small set-asides is often unfavorable in relation to contemporary policies about ecological sustainability (Aune et al. 2005; Angelstam et al. 2011a; Elbakidze et al. 2011). This also means that the majority of PAs in the middle and southern boreal sub-regions are not able to maintain ecological process (Gaston et al. 2008), which are important for biodiversity and other ecosystem services.

Third, the management of the boreal forest landscape needs to be understood. To ensure sufficient habitat quality in the landscape it is crucial to reintroduce natural processes such as forest fire and flooding where appropriate. Conservation management towards landscape restoration can thus contribute to filling the gap between present amounts of habitat and what is needed to satisfy policy goals (Hanski 2011; Mansourian et al. 2006).

Finally, the land-use in the matrix composition around PAs matters. To understand the role of PAs for ecological sustainability, other set-asides at different spatial scales also need to be mapped, and their duration and management regimes understood. First, trees, groups, and strips of trees are left from harvesting within stands (the so-called retention forestry, Vanha-Majamaa and Jalonen 2001; Gustafsson et al. 2012). Second, some stands with high conservation values are considered as woodland key biotopes and are voluntarily set aside, for example, in the context of forest certification schemes (Timonen et al. 2010; Elbakidze et al. 2011). Finally, clusters of stands or entire landscapes are managed for the benefit of different species (Angelstam and Bergman 2004). Key challenges are to measure, aggregate, and assess these efforts in a landscape or an ecoregion so that it is possible to communicate the consequences of the conservation efforts at different spatial scales to different stakeholders (Angelstam and Bergman 2004; Schmitt et al. 2009).

Additionally, we stress that the Aichi target of 17 % PAs refers to the areas that “are conserved through effectively and equitably managed, ecologically representative and well-connected systems of PAs and other effective area-based conservation measures, and integrated into the wider landscape and seascape” (CBD 2011). Thus, while a response indicator such as PA may seem favorable, the pressure on PAs and the surrounding matrix may still be high. In order to fulfill the Aichi target for Europe’s boreal forest it would be useful to formulate biodiversity conservation targets based on analyses of indicators relating to the state of biodiversity, pressures on biodiversity, policy and management responses, and the state of ecosystem services that people derive from biodiversity. Based on these indicators the PA targets are likely to be different for each sub-region and for different countries, and thus the need for landscape and habitat restoration (Angelstam et al. 2011a). Finally, we argue that the development over time of different PA categories in different countries located in the same ecoregion can provide important input to a collaborative learning process within and among countries towards the implementation of internationally agreed policies on ecological sustainability (Angelstam et al. 2011b).

## Electronic supplementary material

Below is the link to the electronic supplementary material.

Supplementary material 1 (PDF 82 kb)
