# Supplementary material for: Protected Area as an Indicator of Ecological Sustainability? A Century of Development in Europe’s Boreal Forest
Source: Ambio. 2013 Mar 10;42(2):201–14. doi: 10.1007/s13280-012-0375-1 (PMC3593037; doi:10.1007/s13280-012-0375-1)
Supplement: Supplementary file 1 — Supplementary material 1 (PDF 82 kb) [file 13280_2012_375_MOESM1_ESM.pdf]

Electronic Supplementary Material

Protected area as an indicator of ecological sustainability? A century of development in Europe's boreal forest

Marine Elbakidze, Per Angelstam, Nikolay Sobolev, Erik Degerman, Kjell Andersson, Robert Axelsson, Olle Höjer, Sandra Wennberg

Table S1 The main units of the European boreal forests based on the explanatory text to the Map of Natural Vegetation of Europe by Bohn et al. (2004)

	Main formations: Mesophytic and hygromesophytic coniferous and mixed broadleaved-coniferous forests			
	Western boreal forest		Eastern boreal forest	
	Spruce forests (*Picea abies, P. obovata, Picea abies x Picea obovata*) with partly *Pinus silvestris,* locally with birch (*Betula pubescens, B. pendula*), alder (*Alnus incana*) or mixed forests		Pine-spruce (*Picea obovata, Pinus sibirica*) and fir-spruce-forests (*Picea obovata, Abies sibirica*), partly with *Betula pubescens*, and *Larix sibirica*	
	Climate and site conditions	Stand structure and floristic composition	Climate and site conditions	Stand structure and floristic composition
Northern boreal forests	Low summer temperature, considerable water supply. Gley podzol and peat soils	Reduced growth, reduced canopy closure and low timber yield. The average height of spruce in mature stands is 15-17 m. Dominant tree layer in the west are *Picea abies*, and *Picea obovata* in the east. Specific species: *Loiseleuria procumbens, Phyllodoce caerulea*. Lichens: *Nephroma arcticum* . Shrubs: *Calluna vulgaris* is common, on dry sites – *Sorbus aucuparia, Juniperus communis* and *Risa acicularis*, on wet site – *Betula nana, Salix lapponum* and *S. phylicifolia.* Birch and aspen are pioneer species after forest fires and clear-cutting.	Low summer temperature, considerable water supply. Loamy-sandy gley podzols and peat-podzol-gley soils.	The tree canopy is low (40-60%) with dominant *Picea obovata*, regular admixtures of birch (*Betula pubescens*) and to a lesser extent *Pinus sibirica* (10-20%). Open and species-poor understorey with *Sorbus aucuparia, Juniperus communis, Rosa acicularis, Betula nana, Alnus fruticosa.*
Middle boreal forests	Moderately warm and moist summers; cold, snow-rich winters. Flat interfluvial plains with glacial and fluvioglcial sediments. Podzolic and wetted soils.	Well developed, usually single-species tree layer. *Picea abies*, *P. abies* x *P. obovata* and/or *P. obovata* with a canopy cover of 70-80%. Average height of fully-grown spruces – 18-20 m. The shrub layer is absent or weakly developed with*Juniperus communis and Sorbus aucuparia* being most frequent. In the western part – *Rosa majalis*,*Rosa acicularis* and *Lonicera pallasii.*	Moderately warm and moist summers; cold, snow-rich winters. Podzols and gley-podzols soils.	Tree species mainly *Picea obovata* and *Abies sibirica*, and on moist sites *Pinus sibirica* and *Larix sibirica.* Widespread species: *Sorbus aucuparia* and Siberian elements – *Sorbus aucuparia* subsp. sibirica, *Rosa acicularis, Lonicera pallasii.* The dominant type of spruce forests with *Vaccinim myrtillus, Linnea borealis, Maianthemum bifolium, Vaccinium vitis-idaea, Luzula pilosa, Orthilia secunda.*

Southern boreal forests	A mild climate, a longer vegetation period. Soils are weakly podzolic. A rough and well structured glacial accumulation relief after being glaciated during the last ice-age.	Stands are dominated by *Picea abies* in the west and *Picea obovata* and transition forms with *Betula pendula*, *B. pubescens* and/or *Populus tremula*. The shrub layer is very open with *Sorbus aucuparia, Corylus avellana, Lonicera xylosteum* and *Daphne mezereum.*	A mild climate, podzoloc, loamy-sandy and loamy soils.	The tree layer is formed by *Picea obovata* and *Abies sibirica*. The shrub layer is well developed and species-rich with nemoral species: *Tilia cordata, Lonicera xylosteum, Viburnum opulus, Frangula alnus, Rosa acicularis* and *R. majalis*. The herb layer has a high degree of coverage and species-rich with typical boreal forests as well as nemoral elements.

Table S2 The sources of data on protected areas in Europe's boreal forest

Published sources

1. Bakka, A., and S. Bakka. 1997. *Особоохраняемые природные территории Нижегородской области* [Specially Protected Natural Areas of Nizhegorodskaya Oblast]. Annotated list: Nizhniy Novgorod (in Russian)

2. Bakka, S., and N. Kiseliova. 2009. *Особоохраняемые природные территории Нижегородской области* [Specially Protected Natural Areas of Nizhegorodskaya Oblast']. Annotated list: Nizhniy Novgorod. (in Russian)

3. Ban'kovskiy, L. (ed.) 1983. *Природные монументы Пермской области.* [Natural Monuments of Permskaya Oblast'] Perm'. (in Russian)

4. Degteva, S. (ed). 2010 *Особоохраняемые природные территориии Республики Коми: результаты GAP-анализа и перспективы развития* [Specially Protected Natural Areas of Komi Republic: outputs of the GAP-analysis and development perspectives] Syktyvkar. (in Russian)

5. Kobyakov, K. (ed). 2011. *Картирование территорий высокой природоохранной ценности на Северо-западе России: Gap-анализ сети охраняемых территорий Мурманскоого, Ленинградского, Архангельского, Вологодского и Карельского регионов и города Санкт-Петербург* [Mapping of High Conservation Value Areas in Northwestern Russia: Gap-Analysis of the Protected Areas Network in the Murmansk, Leningrad, Arkhangelsk, Vologda, and Karelia regions, and the city of Saint-Petersburg] St. Petersburg. (in Russian)

6. Litvinova, E. 2011. Состояние сети охраняемые территорий в Новгородском регионе: история формирования и влияние социальных условий [State of the network of protected areas in the Novgorod region: formation history and the impact of social conditions[. In *Geographic basis for the development of the ecological networks in Russia and Eastern Europe,* 78– 83. Proceedings of the Valdai conference (4-9 April 2011). Moscow: RAS Institute of Geography. (in Russian)
Noskov, G. (ed.). 2004.

7. *Красная книга природы Санкт-Петербурга.Охраняемые территории* [Red Data Book of Nature of Saint-Petersburg. Protected Areas]ю Professional ANO NGO: Saint-Petersburg. (in Russian)

8. Oviosnov, S. (ed). 2009. *Особоохраняемые природные территории Пермской области. Инвентаризация* [Specially Protected Natural Areas of Permskaya Oblast'. An inventory] Kniznyi mir: Perm' (in Russian)

Internet sources

http://kartkatalog.dirnat.no/map_catalog_dataset.asp?datasetid=0&download=yes&language=EN
http://www.eea.europa.eu/data-and-maps/data/nationally-designated-areas-national-cdda-5http://oopt.info Checked 10.09.2012.
http://paslo.ru Checked 10.09.2012
www.duproos.ru/files/gd2010_gl7.doc Checked 10.09.2012
www.duproos.ru/files/gd2010_pril3.doc Checked 10.09.2012.
http://prirodnadzor.mari-el.ru/oopt.html Checked 10.09.2012.
http://www.oopt.aari.ru Checked 10.09.2012.
http://www.biodiversity.ru/kola . Checked 10.09.2012
http://zapoved.ru Checked 10.09.2012.
http://kp.ivanovoobl.ru/deyatelnost_komiteta/osobo_okhranyaemye_prirodnye_territorii.php
http://kp.ivanovoobl.ru/upload/iblock/df3/OOPT http://kp.ivanovoobl.ru/upload/iblock/df3/OOPT 10.09.2012
http://priroda.pskov.ru/oopt-pskovskoi-oblasti
http://www.dvinaland.ru/power/departments/comeco/terrs/ Checked 10.09.2012.
http://www.kirovreg.ru/econom/prres/zakaznik/ Checked 10.09.2012.
http://oopt.kosmosnimki.ru Checked 10.09.2012.
http://forest-karelia.ru/uploads/ff.png Checked 10.09.2012.

9. Soloviova, N. (ed). 2002. *Особоохраняемые природные территории Республики Удмуртия.* [Natural Areas of Udmurtia Republic]. Izhevsk. (in Russian)

10. Stepanitskiy, V., Stishov, M., Troitskaya, N., and A. Troitskiy. 2009. Анализ форм управления и категирий федеральных особоохраняемых природных территорий [Analysis of management forms and categories of Federal Specially Protected Natural Areas] In: *Особоохраняемые природные территории России: современное состояние и перспективы развития* [Specially Protected Natural Areas of Russia: current state and development perspectives], 22–50. Ed. Krever, V., Stishov, M., and I. Onufrenia. WWF Russia: Moscow (in Russian)

11. Tishkov, A. 2002b. Nature protection and conservation. In The physical geography of Northern Eurasia, ed. M. Shahgedanova, 227–245. Oxford: Oxford University Press.

12. Tyusov, A. 2008. *Оценка охранного статуса территории через пространственный анализ распространения редких видов: на примере Тверской области* [Assessing conservation status of a territory through spatial analysis of rare species distribution: an example of Tverskaya Oblast'. PhD thesis] Perm'. (in Russian)
